# Di-μ-chlorido-bis­(chlorido­{*N*′-[phen­yl(pyridin-2-yl-κ*N*)methyl­idene]pyridine-2-carbohydrazide-κ^2^ *N*′,*O*}cadmium)

**DOI:** 10.1107/S1600536814010630

**Published:** 2014-05-17

**Authors:** Mehmet Akkurt, Ali Akbar Khandar, Muhammad Nawaz Tahir, Farhad Akbari Afkhami, Seyed Abolfazl Hosseini Yazdi

**Affiliations:** a Department of Physics, Faculty of Sciences, Erciyes University, 38039 Kayseri, Turkey; b Department of Inorganic Chemistry, Faculty of Chemistry, University of Tabriz, Tabriz, Iran; c Department of Physics, University of Sargodha, Sargodha, Pakistan

## Abstract

The title compound, [Cd_2_Cl_4_(C_18_H_14_N_4_O)_2_], was obtained from the reaction of Cd(NO_3_)_2_·4H_2_O with 2-phenyl­pyridine­keton picolinoyl hydrazone and sodium chloride. Each Cd^2+^ cation is coordinated by two N atoms and one O atom of the tridentate ligand and three chloride anions, forming a distorted CdNOCl_3_ octahedron. Each pair of adjacent metal cations is linked by two bridging chloride ligands, resulting in a dinuclear complex unit. The mol­ecular conformation is stabilized by intra­molecular N—H⋯N and C—H⋯O hydrogen bonds. In the crystal, mol­ecules are linked by nonclassical C—H⋯O and C—H⋯Cl hydrogen bonds into a three-dimensional network. In addition, π–π stacking inter­actions [centroid–centroid distances = 3.777 (2) and 3.631 (2) Å] contribute to the stabilization of the crystal packing.

## Related literature   

For related complexes with similar tridentate ligands, see: Akkurt *et al.* (2012 ▶); Chen *et al.* (2005 ▶); Datta *et al.* (2011 ▶). 
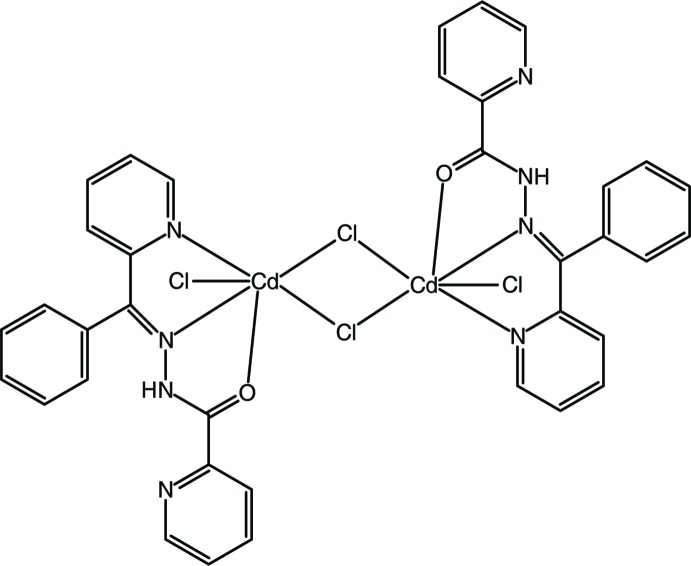


## Experimental   

### 

#### Crystal data  

[Cd_2_Cl_4_(C_18_H_14_N_4_O)_2_]
*M* *_r_* = 971.28
Monoclinic, 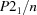

*a* = 15.3215 (4) Å
*b* = 14.4905 (4) Å
*c* = 17.5457 (5) Å
β = 104.667 (1)°
*V* = 3768.49 (18) Å^3^
*Z* = 4
Mo *K*α radiation
μ = 1.46 mm^−1^
*T* = 296 K
0.23 × 0.20 × 0.18 mm

#### Data collection  

Bruker Kappa APEXII CCD diffractometer
Absorption correction: multi-scan (*SADABS*; Bruker, 2005 ▶) *T*_min_ = 0.722, *T*_max_ = 0.769
36565 measured reflections
9369 independent reflections
5625 reflections with *I* > 2σ(*I*)
*R*_int_ = 0.066

#### Refinement  

*R*[*F* ^2^ > 2σ(*F* ^2^)] = 0.044
*wR*(*F* ^2^) = 0.083
*S* = 0.98
9369 reflections
469 parameters
H-atom parameters constrained
Δρ_max_ = 0.47 e Å^−3^
Δρ_min_ = −0.43 e Å^−3^

### 

Data collection: *APEX2* (Bruker, 2009 ▶); cell refinement: *SAINT* (Bruker, 2009 ▶); data reduction: *SAINT*; program(s) used to solve structure: *SHELXS97* (Sheldrick, 2008 ▶); program(s) used to refine structure: *SHELXL97* (Sheldrick, 2008 ▶); molecular graphics: *ORTEP-3 for Windows* (Farrugia, 2012 ▶) and *PLATON* (Spek, 2009 ▶); software used to prepare material for publication: *WinGX* (Farrugia, 2012 ▶) and *PLATON*.

## Supplementary Material

Crystal structure: contains datablock(s) global, I. DOI: 10.1107/S1600536814010630/rz5124sup1.cif

Structure factors: contains datablock(s) I. DOI: 10.1107/S1600536814010630/rz5124Isup2.hkl

CCDC reference: 1001915

Additional supporting information:  crystallographic information; 3D view; checkCIF report

## Figures and Tables

**Table 1:** Hydrogen-bond geometry (Å, °)

*D*—H⋯*A*	*D*—H	H⋯*A*	*D*⋯*A*	*D*—H⋯*A*
N3—H3*A*⋯N4	0.86	2.29	2.646 (4)	105
N7—H7⋯N8	0.86	2.16	2.569 (4)	108
C1—H1⋯O2	0.93	2.53	3.303 (6)	140
C10—H10⋯O1^i^	0.93	2.50	3.261 (6)	139
C36—H36⋯Cl3^ii^	0.93	2.77	3.482 (5)	135

Symmetry codes: (i) 
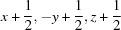
; (ii) 
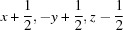
.

**Fig. 1.**
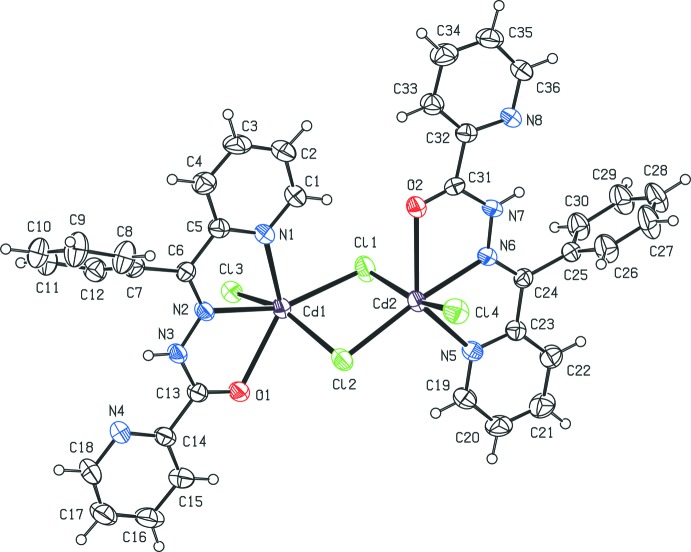
The molecular structure of the title compound with displacement ellipsoids drawn at the 40% probability level.

## supplementary crystallographic information

### 1. Comment

Schiff base complexes have attracted much attention due to their interesting structures and wide potential applications. Recently, the relative unsymmetrical tri- and tetradentate Schiff base ligands and their hydrogenated derivatives have been introduced in coordination chemistry to assemble polymers with beautiful molecular structures. Some organic N-donor ligands are often chosen to fabricate these complexes. In this connection, some complexes with similar tridentate ligands have been studied (Chen *et al.*, 2005; Datta *et al.*, 2011; Akkurt *et al.*, 2012). Herein, we report the structure of a new cadmium complex based on a pyridine based chelating Schiff base ligand. The title compound shows chloride-bridged dinuclear Cd(II) units (Fig. 1). The geometry around each Cd(II) ion is distorted octahedral, in which three positions are occupied by two N atoms and one O atom from the Schiff base ligand, two positions by two bridging chloride anions and the sixth position by one terminal chloride anion. Intramolecular non-classical hydrogen bonds of the type C—H···O and N—H···N are present (Table 1). Non-classical intermolecular hydrogen bonds of type C—H···O and C—H···Cl link complexes into a three-dimensional network (Table 1). In the crystal, π-π stacking interactions also contribute to the stabilization: *Cg*6···*Cg*9 (1 - *x*, 1 - *y*, 1 - *z*) = 3.777 (2) Å, *Cg*7···*Cg*9(-1/2 + *x*, 1/2 - *y*, 1/2 + *z*) = 3.631 (2) Å; where *Cg*6, *Cg*7 and *Cg*9 are the centroids of the N1/C1–C5, N4/C14–C18 and N8/C32–C36 pyridine rings, respectively.

### 2. Experimental

The potentialy tridenatate ligand 2-phenylpyridineketon picolinoyl hydrazone was obtained by condensation of an equimolar mixture of 2-phenylpyridineketon and picolinic acid hydrazide in methanol. The title compound was synthesized by the reaction of a methanolic solution of the ligand and Cd(NO_3_)_2_·4H_2_O in the presence of excess amount of NaCl. The ligand (1 mmol, 0.302 g) and cadmium nitrate (1 mmol, 0.308 g) were placed in the main arm of the branched tube; sodium chloride (2 mmol, 0.117 g) was added to the mixture too. Methanol was carefully added to fill the arms. The tube was sealed and the ligand-containing arm was immersed in an oil bath at 333 K while the branched arm was kept at ambient temperature. After three days, suitable single crystals had deposited in the cooler arm which were isolated, filtered off, washed with acetone and ether and air dried.

### 3. Refinement

The structure was solved by the Patterson method. All H atoms were positioned geometrically with C—H = 0.93, N—H = 0.86 Å, and refined using a riding model with *U*_iso_(H) = 1.2*U*_eq_(C, N).

### Crystal data

**Table:**

[Cd_2_Cl_4_(C_18_H_14_N_4_O)_2_]	*F*(000) = 1920
*M**_r_* = 971.28	*D*_x_ = 1.712 Mg m^−^^3^
Monoclinic, *P*2_1_/*n*	Mo *K*α radiation, λ = 0.71073 Å
Hall symbol: -P 2yn	Cell parameters from 310 reflections
*a* = 15.3215 (4) Å	θ = 3.5–18.2°
*b* = 14.4905 (4) Å	µ = 1.46 mm^−^^1^
*c* = 17.5457 (5) Å	*T* = 296 K
β = 104.667 (1)°	Prism, orange
*V* = 3768.49 (18) Å^3^	0.23 × 0.20 × 0.18 mm
*Z* = 4	

### Data collection

**Table:**

Bruker Kappa APEXII CCD diffractometer	9369 independent reflections
Radiation source: fine-focus sealed tube	5625 reflections with *I* > 2σ(*I*)
Graphite monochromator	*R*_int_ = 0.066
ω scans	θ_max_ = 28.4°, θ_min_ = 1.9°
Absorption correction: multi-scan (*SADABS*; Bruker, 2005)	*h* = −20→17
*T*_min_ = 0.722, *T*_max_ = 0.769	*k* = −19→19
36565 measured reflections	*l* = −23→23

### Refinement

**Table:**

Refinement on *F*^2^	Primary atom site location: structure-invariant direct methods
Least-squares matrix: full	Secondary atom site location: difference Fourier map
*R*[*F*^2^ > 2σ(*F*^2^)] = 0.044	Hydrogen site location: inferred from neighbouring sites
*wR*(*F*^2^) = 0.083	H-atom parameters constrained
*S* = 0.98	*w* = 1/[σ^2^(*F*_o_^2^) + (0.0251*P*)^2^] where *P* = (*F*_o_^2^ + 2*F*_c_^2^)/3
9369 reflections	(Δ/σ)_max_ = 0.002
469 parameters	Δρ_max_ = 0.47 e Å^−^^3^
0 restraints	Δρ_min_ = −0.43 e Å^−^^3^

### Special details

**Table:**

Geometry. Bond distances, angles *etc*. have been calculated using the rounded fractional coordinates. All su's are estimated from the variances of the (full) variance-covariance matrix. The cell e.s.d.'s are taken into account in the estimation of distances, angles and torsion angles
Refinement. Refinement on *F*^2^ for ALL reflections except those flagged by the user for potential systematic errors. Weighted *R*-factors *wR* and all goodnesses of fit *S* are based on *F*^2^, conventional *R*-factors *R* are based on *F*, with *F* set to zero for negative *F*^2^. The observed criterion of *F*^2^ > σ(*F*^2^) is used only for calculating -*R*-factor-obs *etc*. and is not relevant to the choice of reflections for refinement. *R*-factors based on *F*^2^ are statistically about twice as large as those based on *F*, and *R*-factors based on ALL data will be even larger.

### Fractional atomic coordinates and isotropic or equivalent isotropic displacement parameters (Å^2^)

**Table:**

	*x*	*y*	*z*	*U*_iso_*/*U*_eq_	
Cd1	0.21922 (2)	0.30193 (2)	0.59332 (2)	0.0425 (1)	
Cd2	0.21468 (2)	0.45128 (2)	0.41438 (2)	0.0418 (1)	
Cl1	0.23181 (8)	0.27779 (7)	0.44471 (6)	0.0563 (4)	
Cl2	0.15115 (7)	0.45550 (7)	0.53781 (6)	0.0542 (4)	
Cl3	0.20787 (7)	0.13663 (7)	0.62103 (6)	0.0505 (4)	
Cl4	0.24456 (7)	0.61788 (7)	0.40603 (7)	0.0599 (4)	
O1	0.09558 (16)	0.32324 (19)	0.66423 (15)	0.0490 (10)	
O2	0.38776 (16)	0.43618 (19)	0.42115 (15)	0.0518 (10)	
N1	0.3800 (2)	0.3236 (2)	0.63954 (18)	0.0444 (11)	
N2	0.2716 (2)	0.3262 (2)	0.73505 (17)	0.0411 (11)	
N3	0.20746 (19)	0.3262 (2)	0.77667 (17)	0.0445 (11)	
N4	0.0893 (2)	0.3191 (2)	0.8634 (2)	0.0569 (14)	
N5	0.0819 (2)	0.4427 (2)	0.30909 (18)	0.0416 (11)	
N6	0.2485 (2)	0.4326 (2)	0.29130 (17)	0.0393 (11)	
N7	0.3364 (2)	0.4316 (2)	0.28770 (18)	0.0474 (13)	
N8	0.4953 (2)	0.4259 (2)	0.26459 (18)	0.0482 (12)	
C1	0.4346 (3)	0.3258 (3)	0.5914 (3)	0.0551 (17)	
C2	0.5277 (3)	0.3262 (3)	0.6176 (3)	0.0616 (17)	
C3	0.5663 (3)	0.3206 (3)	0.6964 (3)	0.0613 (18)	
C4	0.5109 (3)	0.3168 (3)	0.7475 (2)	0.0544 (16)	
C5	0.4181 (2)	0.3203 (3)	0.7178 (2)	0.0428 (14)	
C6	0.3553 (2)	0.3207 (2)	0.7699 (2)	0.0387 (12)	
C7	0.3922 (2)	0.3131 (3)	0.8569 (2)	0.0414 (14)	
C8	0.4468 (3)	0.3800 (3)	0.8997 (2)	0.0660 (19)	
C9	0.4863 (3)	0.3687 (4)	0.9794 (3)	0.080 (2)	
C10	0.4692 (3)	0.2909 (4)	1.0169 (3)	0.074 (2)	
C11	0.4167 (3)	0.2230 (3)	0.9757 (3)	0.0662 (17)	
C12	0.3767 (3)	0.2335 (3)	0.8955 (2)	0.0543 (17)	
C13	0.1197 (2)	0.3233 (3)	0.7360 (2)	0.0397 (12)	
C14	0.0537 (3)	0.3196 (3)	0.7857 (2)	0.0431 (14)	
C15	−0.0371 (3)	0.3151 (3)	0.7504 (3)	0.0533 (17)	
C16	−0.0959 (3)	0.3084 (3)	0.7988 (3)	0.0675 (19)	
C17	−0.0605 (4)	0.3073 (3)	0.8781 (3)	0.079 (2)	
C18	0.0301 (4)	0.3131 (3)	0.9079 (3)	0.075 (2)	
C19	−0.0018 (3)	0.4465 (3)	0.3178 (3)	0.0534 (17)	
C20	−0.0766 (3)	0.4436 (3)	0.2548 (3)	0.0621 (18)	
C21	−0.0667 (3)	0.4379 (3)	0.1802 (3)	0.0638 (17)	
C22	0.0197 (3)	0.4354 (3)	0.1698 (2)	0.0513 (17)	
C23	0.0922 (2)	0.4377 (2)	0.2349 (2)	0.0381 (12)	
C24	0.1858 (2)	0.4351 (2)	0.2271 (2)	0.0375 (12)	
C25	0.2074 (2)	0.4374 (3)	0.1484 (2)	0.0399 (14)	
C26	0.2287 (3)	0.5202 (3)	0.1180 (3)	0.0651 (19)	
C27	0.2538 (4)	0.5219 (3)	0.0479 (3)	0.081 (2)	
C28	0.2607 (3)	0.4422 (3)	0.0094 (3)	0.071 (2)	
C29	0.2422 (3)	0.3600 (3)	0.0393 (3)	0.0686 (19)	
C30	0.2160 (3)	0.3567 (3)	0.1094 (2)	0.0570 (17)	
C31	0.4021 (2)	0.4331 (2)	0.3562 (2)	0.0403 (12)	
C32	0.4946 (2)	0.4288 (2)	0.3411 (2)	0.0405 (12)	
C33	0.5696 (3)	0.4262 (3)	0.4027 (2)	0.0503 (17)	
C34	0.6528 (3)	0.4181 (3)	0.3849 (3)	0.0640 (19)	
C35	0.6549 (3)	0.4157 (3)	0.3071 (3)	0.0621 (19)	
C36	0.5760 (3)	0.4195 (3)	0.2494 (3)	0.0567 (17)	
H1	0.40890	0.32720	0.53740	0.0660*	
H2	0.56330	0.33020	0.58200	0.0740*	
H3	0.62870	0.31940	0.71550	0.0740*	
H3A	0.22240	0.32810	0.82730	0.0530*	
H4	0.53580	0.31200	0.80150	0.0650*	
H7	0.35000	0.43000	0.24300	0.0570*	
H8	0.45750	0.43390	0.87470	0.0790*	
H9	0.52440	0.41400	1.00720	0.0960*	
H10	0.49350	0.28440	1.07090	0.0890*	
H11	0.40730	0.16900	1.00120	0.0790*	
H12	0.33970	0.18730	0.86800	0.0650*	
H15	−0.05860	0.31640	0.69590	0.0640*	
H16	−0.15790	0.30490	0.77750	0.0810*	
H17	−0.09840	0.30250	0.91180	0.0950*	
H18	0.05250	0.31300	0.96240	0.0900*	
H19	−0.01010	0.45130	0.36830	0.0640*	
H20	−0.13400	0.44560	0.26330	0.0750*	
H21	−0.11680	0.43570	0.13720	0.0760*	
H22	0.02880	0.43220	0.11950	0.0620*	
H26	0.22610	0.57490	0.14510	0.0780*	
H27	0.26600	0.57800	0.02690	0.0980*	
H28	0.27820	0.44370	−0.03770	0.0860*	
H29	0.24710	0.30560	0.01260	0.0830*	
H30	0.20430	0.30020	0.13000	0.0680*	
H33	0.56520	0.42970	0.45450	0.0610*	
H34	0.70580	0.41440	0.42490	0.0770*	
H35	0.70980	0.41160	0.29370	0.0740*	
H36	0.57880	0.41760	0.19710	0.0680*	

### Atomic displacement parameters (Å^2^)

**Table:**

	*U*^11^	*U*^22^	*U*^33^	*U*^12^	*U*^13^	*U*^23^
Cd1	0.0405 (2)	0.0555 (2)	0.0326 (2)	0.0014 (1)	0.0111 (1)	0.0028 (1)
Cd2	0.0438 (2)	0.0499 (2)	0.0342 (2)	−0.0006 (1)	0.0147 (1)	0.0042 (1)
Cl1	0.0807 (8)	0.0498 (7)	0.0450 (6)	0.0084 (5)	0.0281 (6)	0.0044 (5)
Cl2	0.0621 (7)	0.0590 (7)	0.0498 (6)	0.0107 (5)	0.0298 (5)	0.0078 (5)
Cl3	0.0498 (6)	0.0571 (7)	0.0486 (6)	0.0046 (5)	0.0201 (5)	0.0068 (5)
Cl4	0.0628 (7)	0.0495 (7)	0.0708 (8)	−0.0062 (5)	0.0234 (6)	0.0015 (6)
O1	0.0378 (15)	0.074 (2)	0.0358 (16)	0.0041 (14)	0.0105 (13)	−0.0022 (14)
O2	0.0431 (16)	0.079 (2)	0.0346 (16)	0.0045 (14)	0.0123 (13)	0.0039 (14)
N1	0.0399 (19)	0.054 (2)	0.043 (2)	0.0016 (16)	0.0176 (17)	0.0056 (16)
N2	0.0347 (18)	0.060 (2)	0.0305 (17)	−0.0030 (15)	0.0120 (15)	−0.0048 (15)
N3	0.0341 (18)	0.071 (2)	0.0291 (17)	−0.0005 (16)	0.0094 (15)	−0.0035 (16)
N4	0.054 (2)	0.076 (3)	0.046 (2)	0.0109 (19)	0.0227 (19)	0.0145 (19)
N5	0.0349 (18)	0.046 (2)	0.046 (2)	−0.0049 (15)	0.0144 (16)	0.0038 (15)
N6	0.0342 (18)	0.050 (2)	0.0356 (18)	0.0038 (15)	0.0121 (15)	0.0008 (15)
N7	0.0330 (18)	0.074 (3)	0.039 (2)	0.0101 (17)	0.0161 (16)	0.0026 (17)
N8	0.045 (2)	0.063 (2)	0.042 (2)	0.0088 (17)	0.0210 (17)	0.0026 (17)
C1	0.052 (3)	0.067 (3)	0.052 (3)	0.005 (2)	0.024 (2)	0.011 (2)
C2	0.052 (3)	0.075 (3)	0.067 (3)	0.004 (2)	0.032 (3)	0.008 (3)
C3	0.031 (2)	0.073 (3)	0.083 (4)	−0.005 (2)	0.020 (3)	0.002 (3)
C4	0.041 (2)	0.067 (3)	0.052 (3)	−0.003 (2)	0.006 (2)	−0.006 (2)
C5	0.036 (2)	0.050 (3)	0.043 (2)	0.0003 (18)	0.0111 (19)	−0.0006 (19)
C6	0.034 (2)	0.046 (2)	0.034 (2)	−0.0006 (18)	0.0050 (18)	−0.0017 (17)
C7	0.035 (2)	0.047 (3)	0.039 (2)	0.0000 (18)	0.0035 (18)	−0.0024 (19)
C8	0.085 (4)	0.050 (3)	0.050 (3)	−0.012 (2)	−0.007 (3)	0.001 (2)
C9	0.096 (4)	0.084 (4)	0.045 (3)	−0.014 (3)	−0.009 (3)	−0.005 (3)
C10	0.076 (4)	0.097 (4)	0.041 (3)	0.006 (3)	−0.002 (3)	0.008 (3)
C11	0.073 (3)	0.069 (3)	0.058 (3)	0.011 (3)	0.019 (3)	0.024 (3)
C12	0.052 (3)	0.062 (3)	0.049 (3)	−0.006 (2)	0.013 (2)	−0.004 (2)
C13	0.036 (2)	0.045 (2)	0.040 (2)	−0.0004 (18)	0.013 (2)	−0.0019 (18)
C14	0.043 (2)	0.043 (2)	0.049 (3)	0.0041 (19)	0.022 (2)	0.0009 (19)
C15	0.045 (3)	0.058 (3)	0.060 (3)	0.004 (2)	0.019 (2)	−0.006 (2)
C16	0.046 (3)	0.073 (3)	0.093 (4)	0.004 (2)	0.035 (3)	0.005 (3)
C17	0.078 (4)	0.085 (4)	0.095 (4)	0.020 (3)	0.059 (4)	0.027 (3)
C18	0.085 (4)	0.097 (4)	0.056 (3)	0.020 (3)	0.042 (3)	0.019 (3)
C19	0.041 (3)	0.063 (3)	0.061 (3)	−0.006 (2)	0.022 (2)	0.010 (2)
C20	0.034 (2)	0.069 (3)	0.085 (4)	−0.005 (2)	0.018 (3)	0.015 (3)
C21	0.041 (3)	0.075 (3)	0.068 (3)	−0.012 (2)	0.000 (2)	0.007 (3)
C22	0.044 (3)	0.061 (3)	0.046 (3)	−0.005 (2)	0.006 (2)	0.000 (2)
C23	0.038 (2)	0.035 (2)	0.042 (2)	−0.0034 (17)	0.0114 (19)	0.0005 (18)
C24	0.040 (2)	0.037 (2)	0.035 (2)	0.0057 (17)	0.0087 (19)	0.0013 (17)
C25	0.040 (2)	0.046 (3)	0.034 (2)	0.0056 (18)	0.0102 (18)	0.0000 (18)
C26	0.101 (4)	0.045 (3)	0.059 (3)	0.010 (2)	0.038 (3)	0.000 (2)
C27	0.138 (5)	0.058 (3)	0.064 (3)	0.006 (3)	0.055 (3)	0.015 (3)
C28	0.101 (4)	0.077 (4)	0.049 (3)	0.001 (3)	0.043 (3)	−0.003 (3)
C29	0.108 (4)	0.055 (3)	0.053 (3)	0.006 (3)	0.039 (3)	−0.012 (2)
C30	0.081 (3)	0.044 (3)	0.051 (3)	−0.001 (2)	0.026 (2)	0.001 (2)
C31	0.034 (2)	0.043 (2)	0.044 (2)	0.0101 (17)	0.010 (2)	0.0051 (19)
C32	0.035 (2)	0.041 (2)	0.047 (2)	0.0006 (17)	0.013 (2)	0.0002 (18)
C33	0.042 (3)	0.060 (3)	0.049 (3)	−0.003 (2)	0.012 (2)	−0.005 (2)
C34	0.037 (3)	0.081 (4)	0.070 (3)	−0.004 (2)	0.006 (2)	−0.005 (3)
C35	0.043 (3)	0.070 (3)	0.080 (4)	0.005 (2)	0.028 (3)	0.006 (3)
C36	0.053 (3)	0.070 (3)	0.056 (3)	0.010 (2)	0.030 (2)	0.011 (2)

### Geometric parameters (Å, º)

**Table:**

Cd1—Cl1	2.6857 (11)	C16—C17	1.360 (7)
Cd1—Cl2	2.5444 (11)	C17—C18	1.356 (9)
Cd1—Cl3	2.4590 (11)	C19—C20	1.377 (7)
Cd1—O1	2.535 (3)	C20—C21	1.358 (7)
Cd1—N1	2.411 (3)	C21—C22	1.382 (7)
Cd1—N2	2.437 (3)	C22—C23	1.377 (5)
Cd2—Cl1	2.5693 (11)	C23—C24	1.475 (5)
Cd2—Cl2	2.5902 (11)	C24—C25	1.500 (5)
Cd2—Cl4	2.4684 (11)	C25—C26	1.385 (6)
Cd2—O2	2.633 (3)	C25—C30	1.378 (6)
Cd2—N5	2.380 (3)	C26—C27	1.379 (7)
Cd2—N6	2.360 (3)	C27—C28	1.356 (6)
O1—C13	1.219 (4)	C28—C29	1.360 (6)
O2—C31	1.215 (4)	C29—C30	1.388 (6)
N1—C1	1.331 (6)	C31—C32	1.508 (5)
N1—C5	1.350 (5)	C32—C33	1.364 (5)
N2—N3	1.365 (4)	C33—C34	1.392 (7)
N2—C6	1.276 (4)	C34—C35	1.374 (7)
N3—C13	1.354 (4)	C35—C36	1.367 (7)
N4—C14	1.334 (5)	C1—H1	0.9300
N4—C18	1.341 (7)	C2—H2	0.9300
N5—C19	1.331 (6)	C3—H3	0.9300
N5—C23	1.352 (5)	C4—H4	0.9300
N6—N7	1.364 (4)	C8—H8	0.9300
N6—C24	1.283 (4)	C9—H9	0.9300
N7—C31	1.359 (5)	C10—H10	0.9300
N8—C32	1.346 (5)	C11—H11	0.9300
N8—C36	1.332 (6)	C12—H12	0.9300
N3—H3A	0.8600	C15—H15	0.9300
N7—H7	0.8600	C16—H16	0.9300
C1—C2	1.384 (7)	C17—H17	0.9300
C2—C3	1.362 (7)	C18—H18	0.9300
C3—C4	1.383 (6)	C19—H19	0.9300
C4—C5	1.386 (6)	C20—H20	0.9300
C5—C6	1.486 (5)	C21—H21	0.9300
C6—C7	1.492 (5)	C22—H22	0.9300
C7—C12	1.388 (6)	C26—H26	0.9300
C7—C8	1.373 (6)	C27—H27	0.9300
C8—C9	1.386 (6)	C28—H28	0.9300
C9—C10	1.364 (8)	C29—H29	0.9300
C10—C11	1.358 (7)	C30—H30	0.9300
C11—C12	1.394 (6)	C33—H33	0.9300
C13—C14	1.494 (5)	C34—H34	0.9300
C14—C15	1.374 (7)	C35—H35	0.9300
C15—C16	1.389 (7)	C36—H36	0.9300
			
Cl1—Cd1—Cl2	82.27 (3)	N4—C18—C17	123.8 (5)
Cl1—Cd1—Cl3	95.23 (3)	N5—C19—C20	122.5 (4)
Cl1—Cd1—O1	137.62 (6)	C19—C20—C21	120.2 (5)
Cl1—Cd1—N1	91.44 (8)	C20—C21—C22	118.3 (4)
Cl1—Cd1—N2	157.41 (8)	C21—C22—C23	119.2 (4)
Cl2—Cd1—Cl3	152.25 (4)	N5—C23—C22	122.2 (3)
Cl2—Cd1—O1	78.26 (7)	N5—C23—C24	116.4 (3)
Cl2—Cd1—N1	107.44 (7)	C22—C23—C24	121.4 (3)
Cl2—Cd1—N2	105.16 (7)	N6—C24—C23	116.7 (3)
Cl3—Cd1—O1	85.56 (7)	N6—C24—C25	121.2 (3)
Cl3—Cd1—N1	100.24 (7)	C23—C24—C25	122.1 (3)
Cl3—Cd1—N2	87.73 (7)	C24—C25—C30	120.6 (3)
O1—Cd1—N1	130.24 (9)	C26—C25—C30	118.8 (4)
O1—Cd1—N2	64.89 (9)	C24—C25—C26	120.2 (4)
N1—Cd1—N2	66.02 (10)	C25—C26—C27	120.3 (4)
Cl1—Cd2—Cl2	83.70 (3)	C26—C27—C28	120.3 (4)
Cl1—Cd2—Cl4	163.43 (4)	C27—C28—C29	120.3 (5)
Cl1—Cd2—O2	81.98 (7)	C28—C29—C30	120.5 (4)
Cl1—Cd2—N5	97.59 (7)	C25—C30—C29	119.8 (4)
Cl1—Cd2—N6	92.45 (7)	O2—C31—C32	124.6 (3)
Cl2—Cd2—Cl4	98.11 (4)	N7—C31—C32	111.3 (3)
Cl2—Cd2—O2	123.47 (6)	O2—C31—N7	124.1 (3)
Cl2—Cd2—N5	102.82 (8)	N8—C32—C31	115.0 (3)
Cl2—Cd2—N6	169.63 (8)	N8—C32—C33	124.9 (3)
Cl4—Cd2—O2	83.35 (7)	C31—C32—C33	120.2 (3)
Cl4—Cd2—N5	98.05 (8)	C32—C33—C34	117.4 (4)
Cl4—Cd2—N6	88.33 (8)	C33—C34—C35	118.6 (4)
O2—Cd2—N5	133.15 (9)	C34—C35—C36	119.7 (5)
O2—Cd2—N6	65.16 (9)	N8—C36—C35	123.1 (5)
N5—Cd2—N6	68.06 (11)	N1—C1—H1	118.00
Cd1—Cl1—Cd2	92.58 (3)	C2—C1—H1	118.00
Cd1—Cl2—Cd2	95.45 (4)	C1—C2—H2	121.00
Cd1—O1—C13	116.0 (2)	C3—C2—H2	121.00
Cd2—O2—C31	112.3 (2)	C2—C3—H3	121.00
Cd1—N1—C1	122.9 (3)	C4—C3—H3	121.00
Cd1—N1—C5	118.6 (2)	C3—C4—H4	120.00
C1—N1—C5	117.8 (3)	C5—C4—H4	120.00
Cd1—N2—N3	116.8 (2)	C7—C8—H8	119.00
Cd1—N2—C6	120.8 (2)	C9—C8—H8	119.00
N3—N2—C6	121.1 (3)	C8—C9—H9	120.00
N2—N3—C13	118.1 (3)	C10—C9—H9	120.00
C14—N4—C18	115.7 (4)	C9—C10—H10	120.00
Cd2—N5—C19	124.6 (3)	C11—C10—H10	120.00
Cd2—N5—C23	117.7 (2)	C10—C11—H11	120.00
C19—N5—C23	117.6 (3)	C12—C11—H11	120.00
Cd2—N6—N7	119.4 (2)	C7—C12—H12	120.00
Cd2—N6—C24	120.7 (2)	C11—C12—H12	120.00
N7—N6—C24	119.2 (3)	C14—C15—H15	121.00
N6—N7—C31	118.6 (3)	C16—C15—H15	121.00
C32—N8—C36	116.4 (3)	C15—C16—H16	121.00
N2—N3—H3A	121.00	C17—C16—H16	121.00
C13—N3—H3A	121.00	C16—C17—H17	120.00
N6—N7—H7	121.00	C18—C17—H17	120.00
C31—N7—H7	121.00	N4—C18—H18	118.00
N1—C1—C2	123.4 (5)	C17—C18—H18	118.00
C1—C2—C3	118.9 (5)	N5—C19—H19	119.00
C2—C3—C4	118.7 (4)	C20—C19—H19	119.00
C3—C4—C5	119.6 (3)	C19—C20—H20	120.00
N1—C5—C4	121.5 (3)	C21—C20—H20	120.00
N1—C5—C6	116.4 (3)	C20—C21—H21	121.00
C4—C5—C6	122.1 (3)	C22—C21—H21	121.00
N2—C6—C7	124.7 (3)	C21—C22—H22	120.00
C5—C6—C7	119.5 (3)	C23—C22—H22	120.00
N2—C6—C5	115.8 (3)	C25—C26—H26	120.00
C8—C7—C12	118.4 (3)	C27—C26—H26	120.00
C6—C7—C8	121.9 (3)	C26—C27—H27	120.00
C6—C7—C12	119.6 (3)	C28—C27—H27	120.00
C7—C8—C9	121.2 (4)	C27—C28—H28	120.00
C8—C9—C10	119.8 (5)	C29—C28—H28	120.00
C9—C10—C11	120.2 (5)	C28—C29—H29	120.00
C10—C11—C12	120.6 (4)	C30—C29—H29	120.00
C7—C12—C11	119.8 (4)	C25—C30—H30	120.00
O1—C13—N3	123.0 (3)	C29—C30—H30	120.00
O1—C13—C14	122.0 (3)	C32—C33—H33	121.00
N3—C13—C14	115.0 (3)	C34—C33—H33	121.00
N4—C14—C15	124.5 (4)	C33—C34—H34	121.00
C13—C14—C15	119.8 (3)	C35—C34—H34	121.00
N4—C14—C13	115.8 (4)	C34—C35—H35	120.00
C14—C15—C16	117.9 (4)	C36—C35—H35	120.00
C15—C16—C17	118.3 (5)	N8—C36—H36	118.00
C16—C17—C18	119.9 (5)	C35—C36—H36	118.00
			
Cl2—Cd1—Cl1—Cd2	18.37 (4)	N2—N3—C13—O1	−2.1 (6)
Cl3—Cd1—Cl1—Cd2	170.55 (4)	N2—N3—C13—C14	177.6 (3)
O1—Cd1—Cl1—Cd2	81.41 (10)	C18—N4—C14—C15	0.5 (6)
N1—Cd1—Cl1—Cd2	−89.04 (8)	C14—N4—C18—C17	0.3 (6)
N2—Cd1—Cl1—Cd2	−92.78 (19)	C18—N4—C14—C13	−178.2 (4)
O1—Cd1—N1—C5	22.3 (3)	Cd2—N5—C19—C20	178.3 (3)
N2—Cd1—N1—C5	12.2 (3)	Cd2—N5—C23—C22	−177.9 (3)
Cl1—Cd1—N2—N3	−176.23 (15)	Cd2—N5—C23—C24	1.9 (3)
Cl2—Cd1—N2—N3	77.0 (2)	C23—N5—C19—C20	1.4 (6)
Cl1—Cd1—Cl2—Cd2	−18.28 (4)	C19—N5—C23—C24	179.0 (3)
Cl3—Cd1—Cl2—Cd2	−104.82 (7)	C19—N5—C23—C22	−0.8 (5)
O1—Cd1—Cl2—Cd2	−160.43 (7)	N7—N6—C24—C25	−0.3 (5)
N1—Cd1—Cl2—Cd2	70.79 (8)	C24—N6—N7—C31	177.1 (3)
N2—Cd1—Cl2—Cd2	139.93 (8)	Cd2—N6—C24—C23	−7.8 (4)
Cl3—Cd1—N2—C6	88.5 (2)	Cd2—N6—C24—C25	170.9 (2)
O1—Cd1—N2—C6	174.7 (3)	Cd2—N6—N7—C31	5.9 (4)
N1—Cd1—N2—C6	−13.8 (2)	N7—N6—C24—C23	−179.0 (3)
N2—Cd1—N1—C1	−177.4 (3)	N6—N7—C31—O2	−0.7 (5)
Cl1—Cd1—O1—C13	173.2 (3)	N6—N7—C31—C32	178.3 (3)
Cl2—Cd1—O1—C13	−122.4 (3)	C36—N8—C32—C33	0.4 (5)
Cl3—Cd1—O1—C13	80.3 (3)	C32—N8—C36—C35	0.2 (6)
N1—Cd1—O1—C13	−19.4 (3)	C36—N8—C32—C31	−178.2 (3)
N2—Cd1—O1—C13	−9.3 (3)	N1—C1—C2—C3	2.6 (7)
Cl3—Cd1—N1—C1	99.7 (3)	C1—C2—C3—C4	−1.4 (6)
O1—Cd1—N1—C1	−167.4 (3)	C2—C3—C4—C5	−1.1 (6)
Cl3—Cd1—N2—N3	−78.1 (2)	C3—C4—C5—N1	2.7 (7)
O1—Cd1—N2—N3	8.1 (2)	C3—C4—C5—C6	−177.2 (4)
N1—Cd1—N2—N3	179.7 (3)	N1—C5—C6—C7	177.4 (3)
Cl1—Cd1—N1—C1	4.2 (3)	C4—C5—C6—N2	178.5 (4)
Cl2—Cd1—N1—C1	−78.2 (3)	N1—C5—C6—N2	−1.5 (5)
Cl2—Cd1—N2—C6	−116.4 (2)	C4—C5—C6—C7	−2.7 (6)
Cl2—Cd1—N1—C5	111.5 (3)	C5—C6—C7—C8	64.3 (5)
Cl3—Cd1—N1—C5	−70.6 (3)	C5—C6—C7—C12	−111.0 (4)
Cl1—Cd1—N1—C5	−166.2 (3)	N2—C6—C7—C8	−117.0 (4)
Cl1—Cd1—N2—C6	−9.7 (4)	N2—C6—C7—C12	67.8 (5)
Cl2—Cd2—O2—C31	178.2 (2)	C6—C7—C8—C9	−174.9 (4)
N5—Cd2—Cl2—Cd1	115.46 (8)	C6—C7—C12—C11	175.1 (4)
Cl1—Cd2—O2—C31	101.2 (2)	C12—C7—C8—C9	0.5 (6)
N5—Cd2—Cl1—Cd1	−120.13 (8)	C8—C7—C12—C11	−0.4 (6)
N6—Cd2—Cl1—Cd1	171.68 (8)	C7—C8—C9—C10	−1.6 (7)
Cl4—Cd2—O2—C31	−86.5 (2)	C8—C9—C10—C11	2.7 (8)
N5—Cd2—O2—C31	8.3 (3)	C9—C10—C11—C12	−2.7 (7)
N6—Cd2—O2—C31	4.7 (2)	C10—C11—C12—C7	1.5 (7)
Cl2—Cd2—Cl1—Cd1	−17.98 (4)	O1—C13—C14—N4	179.3 (4)
O2—Cd2—Cl1—Cd1	107.19 (6)	N3—C13—C14—C15	−179.1 (4)
Cl1—Cd2—Cl2—Cd1	19.08 (4)	N3—C13—C14—N4	−0.4 (6)
Cl4—Cd2—Cl2—Cd1	−144.32 (4)	O1—C13—C14—C15	0.6 (7)
O2—Cd2—Cl2—Cd1	−56.95 (8)	C13—C14—C15—C16	177.7 (4)
N5—Cd2—N6—N7	177.6 (3)	N4—C14—C15—C16	−0.9 (7)
Cl1—Cd2—N6—C24	103.6 (2)	C14—C15—C16—C17	0.4 (6)
Cl1—Cd2—N5—C19	89.5 (3)	C15—C16—C17—C18	0.3 (7)
Cl2—Cd2—N5—C19	4.3 (3)	C16—C17—C18—N4	−0.7 (7)
Cl4—Cd2—N5—C19	−96.0 (3)	N5—C19—C20—C21	−0.9 (7)
O2—Cd2—N5—C19	175.6 (3)	C19—C20—C21—C22	−0.2 (7)
N6—Cd2—N5—C19	179.1 (3)	C20—C21—C22—C23	0.7 (6)
Cl1—Cd2—N5—C23	−93.6 (2)	C21—C22—C23—C24	179.9 (3)
Cl2—Cd2—N5—C23	−178.8 (2)	C21—C22—C23—N5	−0.3 (6)
Cl4—Cd2—N5—C23	80.9 (2)	C22—C23—C24—C25	4.9 (5)
O2—Cd2—N5—C23	−7.5 (3)	N5—C23—C24—C25	−174.9 (3)
N6—Cd2—N5—C23	−4.0 (2)	N5—C23—C24—N6	3.8 (4)
Cl1—Cd2—N6—N7	−85.3 (2)	C22—C23—C24—N6	−176.4 (3)
Cl4—Cd2—N6—N7	78.2 (2)	N6—C24—C25—C30	90.3 (4)
O2—Cd2—N6—N7	−5.3 (2)	C23—C24—C25—C26	96.0 (4)
O2—Cd2—N6—C24	−176.4 (3)	C23—C24—C25—C30	−91.1 (4)
N5—Cd2—N6—C24	6.4 (2)	N6—C24—C25—C26	−82.6 (5)
Cl4—Cd2—N6—C24	−92.9 (2)	C24—C25—C26—C27	176.1 (4)
Cd1—O1—C13—N3	9.9 (5)	C24—C25—C30—C29	−175.5 (4)
Cd1—O1—C13—C14	−169.8 (3)	C26—C25—C30—C29	−2.5 (6)
Cd2—O2—C31—N7	−4.0 (4)	C30—C25—C26—C27	3.1 (7)
Cd2—O2—C31—C32	177.1 (2)	C25—C26—C27—C28	−2.2 (8)
Cd1—N1—C5—C6	−10.9 (4)	C26—C27—C28—C29	0.5 (8)
Cd1—N1—C5—C4	169.1 (3)	C27—C28—C29—C30	0.1 (8)
C1—N1—C5—C6	178.3 (3)	C28—C29—C30—C25	1.0 (7)
Cd1—N1—C1—C2	−171.4 (3)	O2—C31—C32—N8	−179.9 (3)
C5—N1—C1—C2	−1.0 (6)	N7—C31—C32—C33	−177.6 (3)
C1—N1—C5—C4	−1.7 (6)	O2—C31—C32—C33	1.4 (5)
N3—N2—C6—C5	179.3 (3)	N7—C31—C32—N8	1.0 (4)
Cd1—N2—C6—C5	13.3 (4)	N8—C32—C33—C34	−1.3 (6)
N3—N2—C6—C7	0.5 (5)	C31—C32—C33—C34	177.2 (3)
C6—N2—N3—C13	−173.8 (3)	C32—C33—C34—C35	1.7 (6)
Cd1—N2—N3—C13	−7.3 (4)	C33—C34—C35—C36	−1.2 (6)
Cd1—N2—C6—C7	−165.5 (3)	C34—C35—C36—N8	0.3 (7)

### Hydrogen-bond geometry (Å, º)

**Table:**

*D*—H···*A*	*D*—H	H···*A*	*D*···*A*	*D*—H···*A*
N3—H3*A*···N4	0.86	2.29	2.646 (4)	105
N7—H7···N8	0.86	2.16	2.569 (4)	108
C1—H1···O2	0.93	2.53	3.303 (6)	140
C10—H10···O1^i^	0.93	2.50	3.261 (6)	139
C36—H36···Cl3^ii^	0.93	2.77	3.482 (5)	135

Symmetry codes: (i) *x*+1/2, −*y*+1/2, *z*+1/2; (ii) *x*+1/2, −*y*+1/2, *z*−1/2.
